# The human intestinal bacterium *Eggerthella lenta* influences gut metabolomes in gnotobiotic mice

**DOI:** 10.20517/mrr.2023.65

**Published:** 2024-01-18

**Authors:** Alina Viehof, Sven-Bastiaan Haange, Theresa Streidl, Kristin Schubert, Beatrice Engelmann, Dirk Haller, Ulrike Rolle-Kampczyk, Martin von Bergen, Thomas Clavel

**Affiliations:** ^1^Functional Microbiome Research Group, Institute of Medical Microbiology, University Hospital of RWTH Aachen, Aachen 52074, Germany.; ^2^Department of Molecular Systems Biology, Helmholtz Centre for Environmental Research (UFZ), Leipzig 04318, Germany.; ^3^ZIEL Institute for Food and Health, Technical University of Munich, Freising 85354, Germany.; ^4^Chair of Nutrition and Immunology, Technical University of Munich, Freising 85354, Germany.; ^5^German Centre for Integrative Biodiversity Research (iDiv) Halle-Jena-Leipzig, Leipzig 04103, Germany.; ^6^Institute of Biochemistry, University of Leipzig, Leipzig 04109, Germany.

**Keywords:** Gut microbiota, Oligo-Mouse-Microbiota, *Eggerthella lenta*, gut-liver axis, metabolomics, proteomics

## Abstract

The intestinal microbiota and its metabolites are known to influence host metabolic health. However, little is known about the role of specific microbes. In this work, we used the minimal consortium Oligo-Mouse-Microbiota (OMM12) to study the function of *Coriobacteriia* under defined conditions in gnotobiotic mice. OMM12 mice with or without the addition of the dominant gut bacterium *Eggerthella lenta* (*E. lenta*) were fed with diets varying in fat content and primary bile acids. *E. lenta* stably colonised the mouse caecum at high relative abundances (median: 27.5%). This was accompanied by decreased occurrence of *Akkermansia muciniphila* and *Enterococcus faecalis*, but results did not reach statistical significance in all groups depending on diet and inter-individual differences. Changes in host parameters (anthropometry, blood glucose, and cholesterol) and liver proteomes were primarily due to diet. In contrast, metabolomes in colon content differed significantly between the colonisation groups. The presence of *E. lenta* was associated with elevated levels of latifolicinin C acid and decreased creatine, sarcosine, N,N-dimethylarginine, and N-Acetyl-DL-methionine. In conclusion, *E. lenta* altered specific metabolites in the colon but did not have significant effects on the mice or liver proteomes under the conditions tested due to marked inter-individual differences.

## INTRODUCTION

The intestinal microbiota influences metabolic health^[1,2]^. For instance, there is good evidence that the communities of microbes in our gut alter diet-host interactions, and that they play a causal role in the development and progression of metabolic diseases^[3-6]^. However, there is little data about specific microbes that are important for these processes^[7-9]^. This is partly due to the complexity of gut microbial ecosystems, including multiple types of microorganisms and hundreds of different bacterial species, of which a substantial fraction is still unknown^[10]^. Hence, it is useful to develop and utilize simplified models of the gut microbiota to study the function of specific ecosystem members^[11,12]^.

*Coriobacteriaceae* represents a family of Gram-positive bacteria within the phylum *Actinomycetota* (formerly Actinobacteria). While species within this family have regularly been associated with bacteraemia, they are also important members of the human gut microbiome such as *Atopobium, Collinsella*, *Eggerthella*, and *Slackia* spp^[13]^. *Coriobacteriaceae* was recently split into multiple families within the class *Coriobacteriia*, including *Eggerthellaceae* [represented by the type genus *Eggerthella*, including its type species *Eggerthella lenta* (*E. lenta*), formerly *Eubacterium lentum*], *Atopobiaceae*, and *Coriobacteriaceae*^[14]^. *E. lenta* is a dominant and prevalent member within human gut microbial ecosystems^[15]^. Besides belonging to the core microbiome, strains within this species are functionally versatile; they can metabolise multiple host-derived molecules (e.g., hormones, bile acids, neurotransmitters)^[16-19]^ and oral compounds such as dietary substrates (e.g., polyphenols)^[20,21]^ and drugs (e.g., digoxin)^[22]^. Despite these important functional assets, few studies have investigated the role of *E. lenta* within the ecosystem and its effects on the host under defined conditions.

Using multiple strains of *E. lenta* in monocolonised mice, Alexander *et al*. recently demonstrated the causal role of *E. lenta* in Th17-dependent colitis, which was attributable to the bacterial enzyme cardiac glycoside reductase 2 (Cgr2)^[23]^. Interestingly, the induction of inflammation could be prevented by the addition of arginine to the diet due to the sensitivity of Cgr2 for this substrate, pointing to the importance of diet-microbe interactions for the regulation of host functions. In the context of metabolic health, several descriptive studies have investigated associations between the occurrence of *Eggerthella* spp. and *E. lenta* in human faeces as detected by sequencing and obesity or type 2 diabetes (T2D). Koh *et al*. reported a higher relative abundance in T2D patients (*n* = 33) linked to the ability of the species to produce imidazole propionate^[24]^. Qin *et al*. also reported a positive association between *E. lenta* and T2D (*n* = 170)^[25]^, but the results from these investigations were confounded by metformin treatment^[26]^. With respect to obesity, Yun *et al*. (*n* = 940) reported a negative association between *Eggerthella* and body mass index (BMI)^[27]^, while Fu *et al*. (*n* = 893) found no significant association with BMI but strong positive or negative association with blood triglyceride or high-density lipoprotein (HDL) cholesterol levels^[28]^, respectively. Hence, results on the occurrence of *Eggerthella* spp. and *E. lenta* in metabolic diseases are conflicting and experimental studies are lacking.

In this work, mice colonised with the Oligo-Mouse-Microbiota (OMM12), a bacterial consortium consisting of 12 bacterial isolates from the mouse intestine^[29]^, with or without additional colonisation by *E. lenta*, were compared*.* The mice were fed different diets varying in fat content and supplemented with bile acids due to the link between *Coriobacteriia* and host metabolic conditions. Metabolomes in the colon and proteomes in the liver were studied.

## METHODS

### Cultivation and preparation of gavage solutions

The original aim of this work was to use a consortium of *Coriobacteriaceae* that represent major functional traits of this family. Hence, *Adlercreutzia mucosicola* DSM 19490^T^ (formerly *Enterorhabdus mucosicola*), *Collinsella aerofaciens* DSM 3979^T^, *E. lenta* DSM 2243^T^, and *Lancefieldella parvula* DSM 20469^T^ (formerly *Atopobium parvulum*) were cultured in broth of Wilkins-Chalgren-Anaerobe medium (WCA, Thermo Fisher Scientific) or brain-heart-infusion (BHI, Oxoid). These culture media were supplemented with 0.05 g/mL L-cysteine (Sigma-Aldrich) and 0.02 g/mL 1,4-dithiothreitol (DTT; Sigma-Aldrich) as reducing agents and 2 mg/L phenosafranin (Sigma Aldrich) as a redox potential indicator. To stimulate the growth of *L. parvula,* the medium was supplemented with 0.02% Tween80 (Fisher Scientific). Strains were grown at 37 °C under anoxic conditions (89.3% N_2_, 6% CO_2_, 4.7% H_2_) in Hungate tubes (Dunn Labortechnik GmbH) containing 9 mL of medium.

For colonising germfree mice, microbial suspensions containing the OMM12 strains^[29]^ with or without the *Coriobacteriia* were produced and aliquoted in an anaerobic chamber (MBraun, Garching, Germany; 89.3% N_2_, 6% CO_2_, 4.7% H_2_) and stored at -80 °C. Three frozen caeca from OMM12 mice (kindly provided by Dr. Marijana Basic, Hannover Medical School, Germany) were cut open and the content was mixed with sterile glass beads (2.5 mm in diameter) in 128 mL sterile, anoxic WCA broth containing 40% (vol/vol) glycerol. After homogenisation by vortexing, the suspension was left to stand for a few minutes to sediment debris before the supernatant was collected. For the OMM12 solution without *Coriobacteriia,* the supernatant was mixed with an equal amount of sterile, anoxic WCA broth prior to aliquoting in screw-cap tubes and storage at -80 °C. For the OMM12 stocks with *Coriobacteriia,* overnight cultures of the four strains were counted in duplicate using a Thoma Chamber and a mixture containing ca. 1 × 10^9^ cells/mL of each strain was mixed 1:1 with the OMM12 supernatant containing 40% (vol/vol) glycerol. The cell density of each of the four *Coriobacteriia* strains in the final gavage solution (0.2 mL) was ca. 1 × 10^8^ cells. Aliquots were stored in screw-cap tubes at 80 °C.

### Gnotobiotic mouse experiments

Animal use was approved by the local institution in charge (Regierung von Oberbayern, approval No. 55.2-1-54-2532-156-2013). The animals were housed in the mouse facility of the Technical University of Munich, ZIEL - Institute for Food & Health.

The experimental scheme is presented in Supplementary Figure 1A. Germfree male C57BL/6N mice were associated with 0.2 mL of the freshly thawed microbial mixtures aforementioned via oral gavage three times within one week at the age of five weeks immediately after weaning. During this process, the mice were allocated to cages, ensuring that each colonisation/diet group included mice from different litters housed in multiple cages [Supplementary Figure 1B]. Male mice were used due to their responsiveness to diet-induced obesity. From week 8 onwards, the diet was changed from autoclaved standard chow (Ssniff GmbH) to synthetic control diet (CD) for two weeks. Thereafter, the mice were fed one of the following diets for eight weeks (*n* = 6-8 per group): CD; CD-BA, control diet supplemented with the primary bile acids cholic acid (CA) and chenodeoxycholic acid (CDCA) (each 0.1% w/w); HFD, lard-based high-fat diet (48% energy from fat); HFD-BA, HFD supplemented with CA and CDCA. These diets were used due to the link between *Coriobacteriia*/*E. lenta* and both bile acid metabolism and host metabolic health. Their references and main differences in composition are provided in Supplementary Figure 1C. Before euthanizing the mice with CO_2_, they were fasted for a minimum of 6 h and blood glucose was measured from the tail using the FreeStyle Lite system (Abbott). During necropsy, systemic EDTA plasma was obtained from the central vein (*vena cava*). Contents of the caecum and colon as well as liver lobes were snap frozen. Epididymal, mesenteric, and subcutaneous white adipose tissues were weighed, and their cumulative weight referred to as white adipose tissue (WAT) mass.

### Cholesterol analysis

Cholesterol concentrations in plasma were quantified using the VITROS Chemistry Products CHOL Slides (Ortho Clinical Diagnostics) with the VITROS 350 Chemistry System (Ortho Clinical Diagnostics).

### Microbial DNA isolation

Caecal content from the gnotobiotic mice or the bacterial stocks used for gavage were mixed with 500 μL Tris-EDTA (TE) buffer containing 7.5 mg lysozyme and incubated at 37 °C for 30 min. 50 μL 10% sodium dodecyl sulfate (SDS, Carl Roth) and 300 μg proteinase K (Carl Roth) were added before incubation at 50 °C for 1 h. The samples were then transferred to 2 mL screw-cap tubes containing 500 mg zirconia/silica beads (0.1 mm; Carl Roth), 250 μL 4M Guanidinethiocyanate (Sigma Aldrich), and 500 μL 5% N-laurolylsarcosine (Sigma Aldrich), and they were incubated at 70 °C for 1 h with constant shaking. Mechanical lysis was then performed using a FastPrep®-24 (3 times, 40 s, 6.5 m/s) (MP Biomedicals). Following the addition of 15 mg Poly(vinylpolypyrrolidone) (PVPP, Merck), the samples were vortexed. Then, 5 μL RNase (10 mg/mL, VWR) was added to 500 μL clear supernatant obtained after two subsequent centrifugation steps (15,000 rcf, 4 °C, 3 min) and the samples were incubated at 37 °C with constant shaking for 20 min. The genomic DNA was purified according to the instructions of the Nucleospin gDNA clean-up kit (Macherey Nagel) and eluted in 50 μL TE buffer. DNA quantity and quality were measured with a NanoDrop (Thermo Fisher Scientific).

### High-throughput *16S rRNA* gene amplicon sequencing

The V3/V4 regions of *16S rRNA* genes were amplified (25 cycles; two-step PCR)^[30]^ using primer 341F and 785R^[31]^, including combinatorial dual indexing. Libraries were purified using magnetic beads (Beckman-Coulter) and then pooled in an equimolar manner prior to sequencing in single-end mode on a MiSeq according to the manufacturer’s instructions (Illumina). The UPARSE-based^[32]^ platform IMNGS^[33]^ was used to process the raw sequence reads with the following parameters: barcode mismatches, 1; expected error, 9; quality trimming score, 20; trimming length, 10 nt; min. sequence length, 200 nt; max. sequence length, 300 nt. Operational taxonomic units (OTUs) were clustered at 97% sequence similarity. Relative abundances of each *Coriobacteriia* and the OMM12 species were obtained after BLAST search (97% sequence identity; 80% of the query sequence length; e-value < 1^-25^) against the corresponding reference *16S rRNA* gene sequence. *Beta*-diversity analysis was performed in Rhea^[34]^. All negative controls (DNA extraction and PCR blanks) did not show any contamination (< 100 reads).

### Proteomics

Proteins from liver samples were extracted by adding 800 µL ice-cold lysis buffer [150 mM NaCl, 10 mM Tris (pH 7.2), 0.1% SDS, 1% Triton X-100, 1% deoxycholate, 5 mM EDTA, pH 7.4, cOmplete^TM^ protease inhibitor] to frozen samples, followed by bead milling (10 min, frequency 30) and 1 h incubation on ice. Lysates were centrifuged (12,000 rpm, 15 min, 4 °C), supernatants were recovered, and protein concentration was determined by DC protein assay (BIO-RAD) according to the manufacturer’s instructions.

For each sample, 20 µg protein lysate was used for sample preparation, as described previously^[35]^. Briefly, proteins were reduced with TCEP [tris(2-carboxyethyl)phosphine hydrochloride] and alkylated with iodoacetamide (IAA). A protein clean-up was conducted using paramagnetic beads in 70% ethanol and acetonitrile (ACN) without acidification of the samples, as described previously^[35]^. Proteins were then proteolytically cleaved using Trypsin (Promega) in a 1:50 ratio (trypsin:protein) for 16 h at 37 °C. Next, samples were labelled using 5× tandem mass tags (TMT, TMT-10plex, Thermo Scientific, USA) for 1 h at room temperature, followed by adding 1 µL 5% (v/v) hydroxylamine in 100 mM TEAB to quench the labelling. Samples were combined into 7 mixes, each containing 1 replicate of the different treatments. After peptide clean-up with 100% ACN, peptides were eluted in 2 fractions using 87% ACN in 10 mM ammonium formate (pH 10, Sigma Aldrich) for fraction 1 and 2% dimethylsulfoxide (DMSO, Sigma Aldrich) for fraction 2.

LC-MS/MS analysis was then performed using an UltiMate 3000 RSLCnano system (Dionex, USA), coupled online to a Q Exactive HF mass spectrometer (Thermo Fisher Scientific, USA) by a chip-based electrospray ionisation source (TriVersa NanoMate, Advion, USA), as previously described^[35,36]^. First, peptides were loaded on a trapping column with 5 μL/min by using 98% water/2% ACN/0.05% trifluoroacetic acid (Acclaim PepMap 100 C18, 3 μm, nanoViper, 75 μm × 5 cm, Thermo Fisher Scientific, Germany) and peptides were then separated on an analytical column (Acclaim PepMap 100 C18, 3 μm, nanoViper, 75 μm × 25 cm, Thermo Fisher, Germany). A 150-min non-linear gradient was applied, starting from 0.1% formic acid in water to 80% ACN/0.08% formic acid in water. From each MS, the top 15 precursor ions were selected with an isolation window of 0.7 m/z.

MS raw data were processed against the *Mus musculus* UniProtKB reference proteome from 16th November 2021 using ProteomeDiscoverer 2.2 with carbamidomethylation. TMT are set as fixed, and methionine oxidation and acetylation of protein N-terminus are set as variable modifications. Reporter ion intensities were corrected according to the manufacturer’s instructions. A total of 1,914 proteins were identified. Samples were normalised against a pool control containing a mixture of all samples included in each TMT mix. Samples were filtered for proteins quantified in at least four replicates per condition, resulting in 1,710 proteins.

### Metabolomics

Samples were prepared, measured, and analysed as described by Pedersen *et al*.^[37]^, using a method adapted from Krause *et al*.^[38]^. The luminal content in the entire colon was mixed with 5× ACN:H_2_O solvent and 4 steel beads for subsequent extraction in the bead mill (10 min, 30 Hz). After centrifugation (14,000 rpm, 2 min), 100 µL supernatant was added to 500 µL MeOH:ACN:H_2_O (2:3:1) and vortexed thoroughly for 5 min. After 5 min of sonication, samples were centrifuged (14.000 rpm, 5 min), 550 µL transferred to a new tube, evaporated to dryness (SpeedVac, Eppendorf), and kept at -80 °C until further use. Prior to analysis, samples were resuspended in 100 µL 0.1% FA and 1% ACN in water. Samples measured in positive and negative ionisation modes were further diluted 1:10.

For measurement, 10 or 5 µL (positive and negative ionisation, respectively) of each extract was injected into a HPLC system coupled online with a 6546 UHD Accurate-Mass Q-TOF (Agilent Technologies). Metabolites were separated with an Agilent Zorbax Eclipse Plus C18 column (2.1 × 100 mm, 1.8 µm) equipped with a matching pre-column (2.1 × 50 mm, 1.8 µm). The autosampler was kept at 5 °C and column oven set to 45 °C. Separation was achieved using a binary solvent system of A (0.1% FA in water) and B (0.1% FA in ACN). The gradient was as follows: 0-5.5 min: 1% B; 5-20 min: 1%-100% B; 20-22 min: 100% B; 22-22.5: 100%-1% B; 22.5-25 min: 1% B. Metabolites were eluted at a constant flow rate of 0.3 mL/min with the autosampler at 5 °C and column oven at 45 °C. Eluted compounds were measured with the QTOF operated in centroid mode. Full scan data was generated with a scan range of 50-1,000 m/z in both ionisation modes. Out of the survey scan, the two most abundant precursor ions with charge state = 1 were subjected to fragmentation. The dynamic exclusion time after two acquired spectra was set to 0.1 min.

The spectral data (.d files) were imported into the Progenesis QI software (Non-Linear Dynamics, Milford, MA, USA). The two ionisation modes were analysed separately. The adduct ions were [M+H], [M+H-H2O] for positive mode and [M-H], [M-H2O-H] and [M+FA-H] for negative mode. Chromatograms were aligned using an automatically chosen reference chromatogram from the dataset. The following software-guided peak-picking tool resulted in a data matrix including retention time, mass-to-charge ratio, and corresponding normalised peak area. For the in-silico database search, the Fecal Metabolome Database was used as a resource. After exporting the results (compound measurement and putative identifications) for all measured compounds, the data was further processed by only keeping peaks that were putatively identified with an MS2 fragment spectrum and a minimum Progenesis score of 40. Only those annotations with the highest Progenesis score were kept if peaks were annotated to multiple compounds.

### Statistics

For microbiota data, statistics were calculated using Rhea^[34]^ in R or using GraphPad Prism (Graphpad Software, Inc) to perform the specific tests mentioned throughout the text and figures. For proteomics, after log2-transformation, fold changes were calculated relative to the respective controls with adjusted *P*-values calculated using the Benjamini-Hochberg method. Proteins were considered significantly altered with an adjusted *P*-value ≤ 0.05. The analysis was conducted in R-3.5.0 using the following packages: mixOmics^[39]^, ggplot2^[40]^, qpcR^[41]^, corrplot^[42]^, PerformanceAnalytics^[43]^, calibrate^[44]^, dendsort^[45]^, dendextend^[46]^, ComplexHeatmap^[47]^, limma^[48]^, plyr^[49]^, reshape2^[50]^, xlsx^[51]^, DEP^[52]^, ggsci^[53]^, ggpubr^[54]^, pheatmap^[55]^, circlize^[56]^. Principal component analysis of metabolome peaks was done in R with the significance of group differences calculated by PERMANOVA using the adonis2 function in the R package vegan^[57]^. Differences in annotated metabolites were calculated in R using a Kruskal-Wallis test with correction for multiple testing by Benjamini-Hochberg method followed by post hoc Dunn’s test for pairwise comparisons.

## RESULTS

### *E. lenta* colonised the intestine of OMM12 mice

Germfree mice were colonised with cryopreserved caecal suspensions of OMM12 mice with or without *Coriobacteriia*. At the end of the experiment (18 weeks of age; ca. 12 weeks of colonisation), the presence of each species in caecum was evaluated by *16S rRNA* gene amplicon sequencing, which delivered 13,202 ± 4,266 high-quality, chimera-checked sequences per sample. Of the four *Coriobacteriia*, only *E. lenta* colonised the intestine.

**Figure 1:**
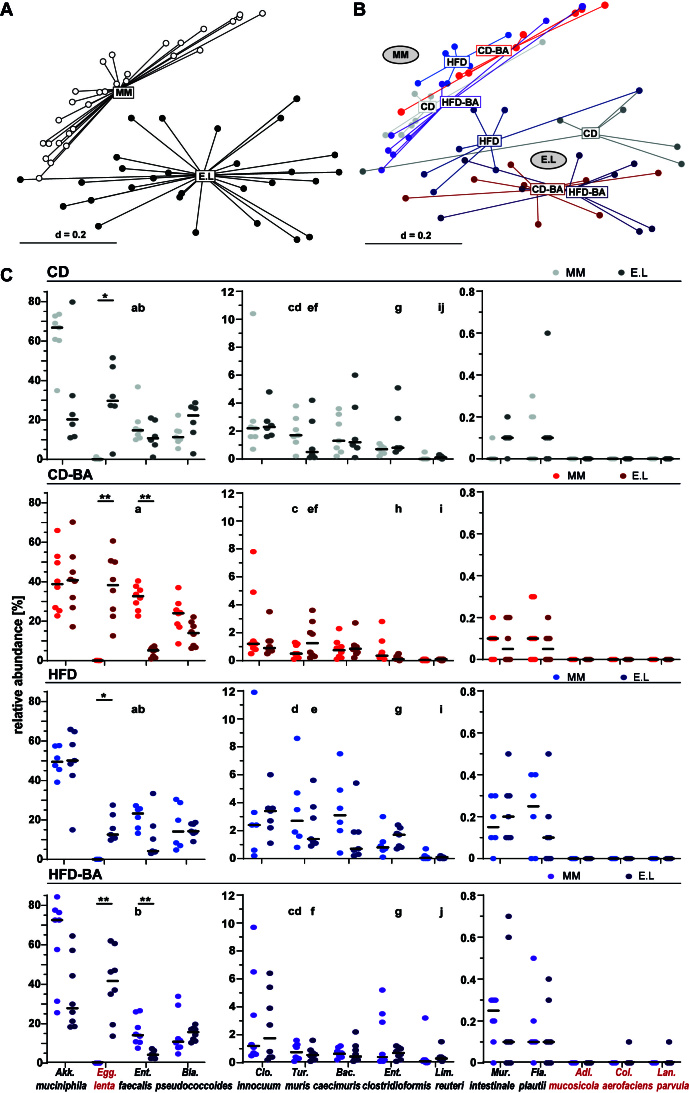
*E. lenta* colonised the caecum of OMM12 mice. (A) Beta-diversity analysis of colonisation groups shown as a multi-dimensional scaling plot of generalised UniFrac distances (*P* = 0.001; PERMANOVA); (B) Same as in panel (A), visualised per colonisation/diet groups (*P* = 0.001; PERMANOVA); (C) Bacterial composition in caecum of gnotobiotic mice (*16S rRNA* gene amplicon sequencing). Data plotted as dots representing individual mice; black bars indicate median values. *B. animalis* and *A. muris* were not detected. Statistics: strains detected in > 50% of the mice in at least one diet group were compared statistically using Mann-Whitney tests with Benjamini-Hochberg adjustment. Stars indicate significant differences between colonisation groups (*adj. *P* < 0.05; **adj. *P* < 0.01; including correction within each diet group; *n* = 11 tests). Different letters indicate significant differences between the corresponding diets for each strain (correction of *P*-values for each strain individually; *n* = 12 tests). A previous version of this figure was published in the PhD thesis by co-author Theresa Streidl^[58]^. OMM12: Oligo-Mouse Microbiota; MM: mice colonised with the mouse synthetic community OMM12; E.L: mice colonised with OMM12 and *Coriobacteriia*; CD: control diet; CD-BA: control diet supplemented with 0.2% primary bile acids; HFD: lard-based high-fat diet; HFD-BA: HFD supplemented with 0.2% primary bile acids.

*Beta*-diversity analysis based on generalised UniFrac distances revealed that the microbial communities mainly differed due to the presence or absence of *E. lenta* (*P* = 0.001, PERMANOVA; Figure 1A). Effects of the diets (BAs and HFD) on the overall phylogenetic makeup were more pronounced in the mice colonised with *E. lenta*, reaching statistical significance only in the case of BA supplementation in HFD (E.L HFD *vs.* E.L HFD-BA, adj. *P* = 0.0482) [Figure 1B]. BAs also affected microbiota profiles in the caecum of OMM12 mice (MM CD *vs.* MM CD-BA, adj. *P* = 0.028). All other pairwise comparisons between diet groups were not statistically significant (adj. *P* > 0.05).

All four *Coriobacteriia* species were detected in the gavage solution used to colonise the mice: *E. lenta* (6.3% relative abundance), *L. parvula* (4.8%), *C. aerofaciens* (3.5%), *A. mucosicola* (1.6%), OMM12 strains (83.9%) [Supplementary Figure 2]. In contrast, only *E. lenta* was detected in mice at a median relative abundance of 27.5%, without significant differences between the diets due to marked inter-individual differences [Figure 1C]. Similar results were obtained in small intestinal content, i.e., *E. lenta* was the only *Coriobacteriia* detected (data not shown).

Ten of the twelve OMM12 strains were detected by sequencing in the gavage solutions [Supplementary Figure 2]. *Bifidobacterium animalis* DSM 26074 (= YL2) and *Acutalibacter muris* DSM 26090^T^ (= KB18) were not detected, as reported in previous mouse studies^[29,59]^. The same ten OMM12 strains were detected in the mouse caecal contents [Figure 1C]. *Akkermansia muciniphila* DSM 26127 (= YL44) was the most dominant species (> 30% relative abundance in most mice), followed by *Enterococcus faecalis* DSM 32036 (= KB1) and *Blautia pseudococcoides* DSM 26115 (= YL58) (both >10%), and *Clostridium innocuum* DSM 26113 (= I46), *Turicimonas muris* DSM 26109 (= YL45), *Bacteroides caecimuris* DSM 26085 (= I48), and *Enterocloster clostridioformis* DSM 26114 (= YL32) (all < 4%). *Limosilactobacillus reuteri* DSM 32035 (= I49), *Muribaculum intestinale* DSM 28989 (= YL27) and *Flavonifractor plautii* DSM 26117 (= YL31) occurred at low relative abundance (< 1%). The presence of *E. lenta* was linked to a decrease in levels of *Ent. faecalis*, which reached statistical significance in animals fed the two diets containing BAs. Relative abundances of *Akk. muciniphila* decreased in CD and HFD-BA mice in the presence of *E. lenta*, but the results did not reach statistical significance. Colonisation by *E. lenta* did otherwise not lead to substantial changes in the microbial communities.

### *E. lenta* did not significantly alter host parameters and liver proteomes

**Figure 2:**
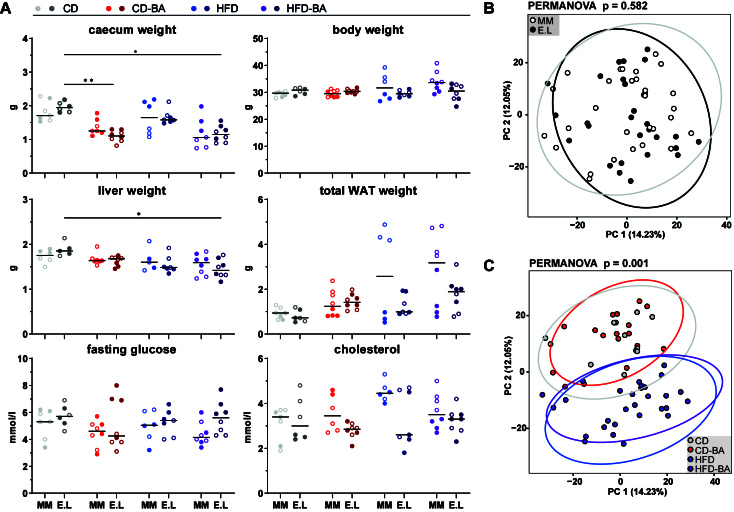
Colonisation with *E. lenta* had no significant effects on the host. (A) Weight of the caecum, body, liver, and total WAT as well as blood levels of fasting glucose and cholesterol. Data plotted as dots representing individual mice. Within each group, mice housed in different cages are indicated with empty or filled circles. Black bars show median values. Statistics: Kruskal-Wallis test followed by Dunn’s test for multiple comparisons. Stars indicate significant differences between diets (*adj. *P* < 0.05; **adj. *P* < 0.01); (B) PCA of liver proteomes per colonisation group (*P* = 0.582; PERMANOVA); (C) Same as in panel (B), shown per diet groups (*P* = 0.001; PERMANOVA). Panel (A) of this figure was published in the PhD thesis by co-author Theresa Streidl^[58]^. WAT: White adipose tissue; PCA: principal component analysis; MM: mice colonised with the mouse synthetic community OMM12; E.L: mice colonised with OMM12 and Coriobacteriia; CD: control diet; CD-BA: control diet supplemented with 0.2% primary bile acids; HFD: lard-based high-fat diet; HFD-BA: HFD supplemented with 0.2% primary bile acids.

Weight of the caecum, body, liver, and total WAT as well as fasting blood glucose and plasma cholesterol levels were not significantly altered by *E. lenta* colonisation [Figure 2A]. Caecum weight was significantly influenced by diet in *E. lenta*-colonised mice; BA supplementation was associated with smaller caeca. Additional colonisation-specific effects due to diet included: (i) highest body weight and cholesterol levels in OMM12 mice fed the HFD-BA and HFD, respectively, although results did not reach statistical significance; (ii) low liver weight in *E. lenta*-colonised mice fed the HFD-BA diet.

Principal component analysis (PCA) of liver proteomes showed no significant differences between colonisation groups (*P* = 0.582, PERMANOVA) [Figure 2B]. In contrast, the different diets led to significant differences in the proteome (*P* = 0.001, PERMANOVA) [Figure 2C]. When comparing fold-changes of single proteins between OMM12 and *E. lenta*-colonised mice for each of the four diet groups, no significantly different proteins were identified. In contrast, significant effects were triggered by the diet, especially fat content [Supplementary Figure 3]. Data on individual proteins and differences for all comparisons are available in Supplementary Table 1. When comparing the HFD-fed mice to those fed the corresponding diet without additional fat (i.e., HFD *vs*. CD and HFD-BA *vs*. CD-BA), consistent changes were observed in both colonisation groups. Fatty acid-binding protein 5 (UniProtKB ID: Q05816) had the highest negative Log2(FC) (-4.23 to -4.84). Cytoplasmic aspartate aminotransferase (UniProtKB ID: P05201), ATP-citrate synthase (UniProtKB ID: Q3V117), and thyroid hormone-inducible hepatic protein (UniProtKB ID: Q62264) were consistently under the top-ten downregulated proteins in the HFD groups. Cholesteryl ester hydrolase (UniProtKB ID: Q9Z0M5) was consistently under the top-ten upregulated proteins in the HFD groups [Log2(FC) 3.28 to 4.69]. BA supplementation also led to significant changes in liver proteomes. In *E. lenta*-colonised mice fed the CD-BA diet, peroxisomal acyl-coenzyme A oxidase 1 (UniProtKB ID: Q9R0H0) was the most substantially downregulated protein, while corticosteroid 11-beta-dehydrogenase isozyme 1 (UniProtKB ID: P50172) was strongly upregulated.

### *E. lenta* colonisation affected metabolomes in the colon

Next, we examined the functional effect of *E. lenta* colonisation in the distal gut using non-targeted metabolomics. A total of 381 and 576 metabolite peaks were identified in the positive and negative ionisation modes, respectively. Of those metabolite peaks, 71 and 87 metabolites could be annotated, respectively. The peak intensity for all annotated metabolites is provided in Supplementary Tables 2 and 3. Heatmaps with individual z-scores for all annotated metabolites are provided in Supplementary Figures 4 and 5.

**Figure 3:**
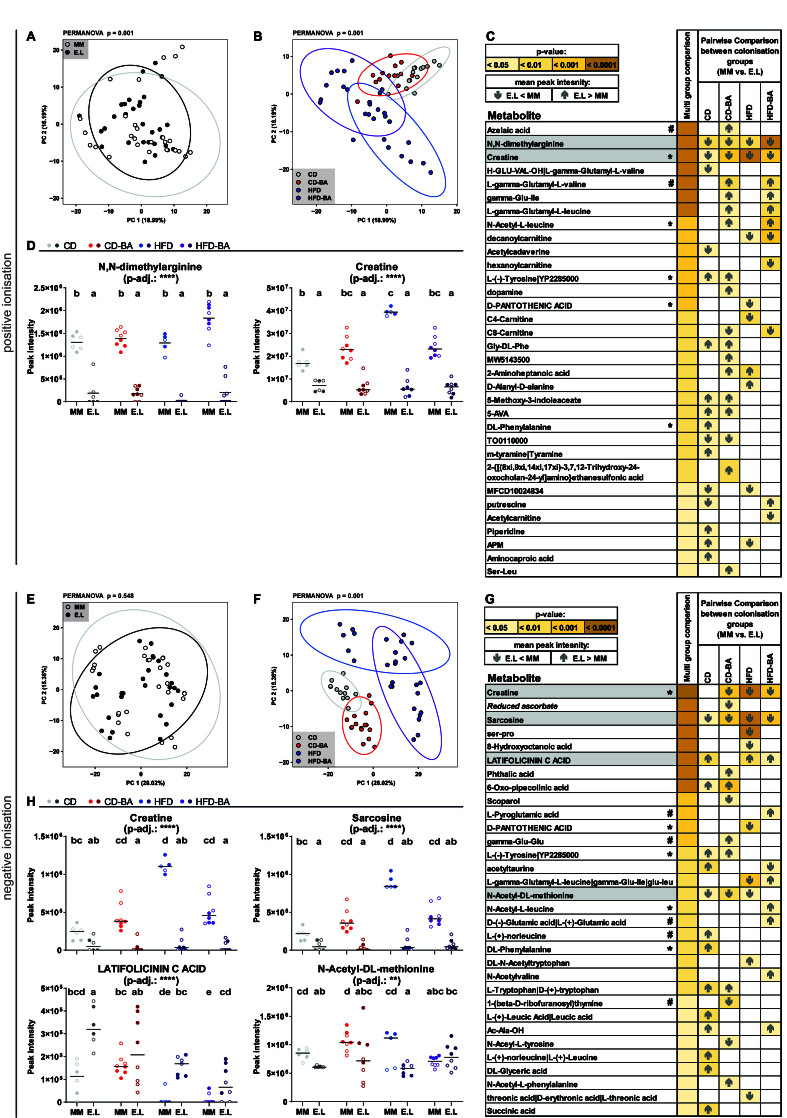
Colonisation with *E. lenta* significantly changed metabolomes in the colon. PCA of all identified metabolite peaks detected in positive ionisation according to (A) colonisation, and (B) diet; (C) Annotated metabolites with significant differences between E.L and MM mice within at least one dietary group as measured in positive ionisation mode. Individual values for the metabolites in grey boxes are shown in panel (D). The symbols after metabolite names indicate those detected in the two ionisation modes and statistically significant in both (*) or only in the specific ionisation mode (#). The strength of significance is shown with a colour gradient (pale yellow to brown). The first coloured column indicates significance (adjusted *P*-values) according to multi-group comparisons (Kruskal-Wallis with Benjamini-Hochberg adjustment). The remaining columns indicate the results of Dunn’s test for post hoc comparisons between E.L and MM mice per diet. The grey arrows indicate the direction of changes as specified in the figure legend; (D) Peak intensity of the individual metabolites showing significant pairwise comparisons between the two colonisation groups in at least 3 of the 4 diets. Dots represent the values for individual mice; black bars indicate median values; within each group, mice housed in different cages are indicated with empty or filled circles. Statistics: **adj. *P* < 0.01, ****adj. *P* < 0.0001, Kruskal-Wallis; for each metabolite, different letters indicate significance between the corresponding groups (Dunn’s test); (E-H) Same as in panels (A-D) for the negative ionisation mode. PCA: Principal component analysis; E.L: mice colonised with OMM12 and *Coriobacteriia*; MM: mice colonised with the mouse synthetic community OMM12; CD: control diet; CD-BA: control diet supplemented with 0.2% primary bile acids; HFD: lard-based high-fat diet; HFD-BA: HFD supplemented with 0.2% primary bile acids; OMM12: Oligo-Mouse Microbiota.

PCA of all detected metabolite peaks revealed significant differences between the colonisation groups in the positive (*P* = 0.001, PERMANOVA) but not negative (*P* = 0.548) ionisation mode [Figure 3A and E]. Clear significant differences (*P* = 0.001) in the overall metabolite landscape were observed between mice fed the different diets, independent of the ionisation mode [Figure 3B and F]. The occurrence of single annotated metabolites in the colon of *E. lenta*-colonised *vs*. OMM12 mice within each diet group was then compared using a Kruskal-Wallis test followed by pairwise comparisons (Dunn’s test) [Figure 3C and G]. In total, 33 (positive mode) and 32 (negative mode) of the annotated metabolites showed significant changes due to colonisation in at least one diet group. Interestingly, two (positive mode) and four (negative mode) metabolites were characterised by consistent changes between the mice across multiple diets (≥ 3) (Figure 3D and H; marked in grey in Figure 3C and G). While latifolicinin C acid was the only metabolite that was elevated in *E. lenta*-colonised *vs*. OMM12 mice (CD, HFD, and HFD-BA diet), creatine (detected in both ionisation modes), sarcosine, N,N-dimethylarginine, and N-Acetyl-DL-methionine were significantly reduced in *E. lenta*-colonised mice.

## DISCUSSION

In this work, we investigated the effects of the dominant human gut bacterial species *E. lenta* on microbial communities and on the host under controlled conditions in gnotobiotic mice.

The three *Coriobacteriia* species *C. aerofaciens*, *L. parvula*, and *A. mucosicola* were not detected by amplicon sequencing. While we cannot exclude that they formed subdominant populations, *E. lenta* was a clear dominant member of the synthetic community in the two gut regions tested. Previous work highlighted that the host origin of strains influences the ability to colonise the mouse intestine^[60,61]^. This may apply to *C. aerofaciens*, as the strain used (type strain) originates from the human gut. However, previous work reported successful colonisation of mice with the same strain^[62,63]^. Moreover, *E. lenta*, the type strain of which also originates from the human gut (rectal tumor), did colonise the mice in our experiment. Hence, other factors such as synthetic community composition or differences in the diet likely influenced species engraftment. *A. mucosicola* was not detected in the present study, although the type strain originates from the mouse gut and it previously colonised mice as part of an extended version of OMM with 19 strains, albeit at low relative abundance^[11]^. Reasons why 3 of 4 *Coriobacteriia* did not colonise the mouse intestine are unclear. As we used only high-throughput amplicon sequencing for detection, we cannot exclude that shotgun sequencing or *16S rRNA* gene-targeted qPCR would have enabled detection of the other *Coriobacteriia*, possibly as members of subdominant populations. Nonetheless, the two groups of mice with distinct synthetic community profiles due to *E. lenta* as a dominant member allowed us to study the effects of presence/absence of this species on all other readouts (anthropometry, liver proteomes, colon metabolomes).

Although *E. lenta* has important metabolic functions and colonised at high relative abundances, its colonisation did not result in strong effects on the investigated host parameters. Hence, we could not experimentally confirm previous observational data linking *Coriobacteriia* and *E. lenta* to host metabolism. We observed a trend towards lower blood cholesterol levels in *E. lenta*-colonised *vs*. OMM12 mice across the different diet groups; however, this was not statistically significant. Martinez *et al.* reported a high positive association between the occurrence of *Coriobacteriia* (as measured by pyrosequencing of *16S rRNA* gene amplicons) and non-HDL plasma cholesterol levels after dietary intervention in a hamster model of hypercholesterolemia^[64]^. However, the underlying species were unclear. In 2011, Claus *et al.* reported a strong and positive association between the occurrence of *E. lenta* and hepatic triglyceride levels following 20 days of reconventionalisation of 8-week-old female germfree mice^[65]^. In our study, liver proteomic data did not hint at major changes in liver functions due to *E. lenta*. In contrast, fat and primary bile acids in the diet had a significant influence on liver proteomes. For example, proteins involved in the arginine biosynthesis pathway were downregulated in mice fed diets with high amounts of fat.

Marked inter-individual differences between mice within some of the dietary groups were observed, which may have masked effects due to colonisation; this is an important limitation of this study. We specifically used an experimental design that included multiple litters of mice and two cages per group to account for these potential confounding factors. While the clear split between mice within the MM/HFD groups in terms of WAT mass and body weight was linked to cage effects, this was not the case, for example, regarding cholesterol in the E.L/HFD group, fasting glucose in the E.L/CD-BA group, and WAT mass in the E.L/HFD-BA group. The two diets enriched in fat changed multiple parameters significantly, including proteomes in the liver and metabolomes in the gut of all mice, elevated body weight (up to +10 g) and white adipose tissue (up to +5 g) in several mice, and increased blood cholesterol in OMM12 mice. Hence, there is no concern about the diets themselves (e.g., composition) or the success of the feeding protocol. Reasons behind the observed inter-individual differences are unclear, but may include differences within a single cage, e.g., differing feed access due to rivalry/hierarchy between male mice.

Liver weight was reduced significantly in mice colonised with *E. lenta* and fed primary bile acids in the HFD. Differences between the other groups were not significant. We observed a similar effect of bile acids being associated with decreased liver weights in other experiments; the mechanism underlying this effect requires further investigation and is out of the scope of this study. With respect to metabolic responses of the mice altogether, two factors are worth noting: (i) mice were fed for 8 weeks only, with a HFD containing 48% energy content from fat (compared with 60% in other studies); the development of fatty liver in C57BL/6N mice usually takes longer than 8 weeks (e.g., 16 weeks of feeding); (ii) the mice were colonised with the synthetic community OMM12. While this allowed us to specifically study the effects of *E. lenta* under controlled conditions, results cannot be compared directly to known effects of HFDs in normally colonised mice.

Colonisation with *E. lenta* altered the levels of specific metabolites in the mouse colon: latifolicinin C acid was elevated, while creatine, sarcosine, N,N-dimethylarginine, and N-Acetyl-DL-methionine were reduced. It was remarkable that these changes were consistent across all groups of mice, as they were fed very different diets (exceptions were latifolicinin C acid and N-Acetyl-DL-methionine, which were not significantly different in mice fed the CD-BA and HFD-BA diet, respectively). Multiple other metabolite changes were observed, but were dependent on the diet fed to the mice. The elevated levels of latifolicinin C acid observed in *E. lenta*-colonised mice are in agreement with the literature, as latifolicinin C acid is a metabolite of tyrosine degradation, which can be produced by multiple gut bacteria, including *E. lenta*^[66,67]^. The decreased colonic levels of creatine suggest that *E. lenta* can degrade it. Gut bacteria have been reported to express enzymes involved in creatinine and creatine degradation^[68]^. The gene encoding for creatininase, which catalyses the reversible conversion of creatinine to creatine, was detected in the genome of *E. lenta* (GCA_000024265.1_01172, WP_009304706.1). Creatine can be further metabolised to urea, as well as sarcosine which can be further degraded to glycine^[68]^. Sarcosine was also significantly decreased in *E. lenta*-colonised mice. However, genes encoding the enzymes that catalyse these reactions were not found in the *E. lenta* genome. N,N-dimethylarginine was also significantly decreased in *E. lenta*-colonised mice. It is an analogue of L-arginine, a known substrate of *E. lenta*^[69]^, and it can be metabolised to dimethylamine and L-citrulline by dimethylarginine dimethylaminohydrolase (DDAH)^[70]^. The gene encoding for this enzyme was detected in the *E. lenta* genome (GCA_000024265.1_02955). Elevated plasma levels of N,N-dimethylarginine have been observed in humans with hypercholesterolemia^[71]^, diabetes mellitus^[72]^, atherosclerosis^[73]^, hypertension^[74]^, chronic heart failure^[75]^, and chronic renal failure^[76]^. In our data, the colonic content of N,N-dimethylarginine was markedly reduced in *E. lenta*-colonised mice. Citrulline, the potential product of N,N-dimethylarginine, was detected in positive ionisation mode, but it was not significantly different between the colonisation groups. In other studies, citrulline was found to be produced and further metabolised by *E. lenta*^[69,77]^.

In conclusion, this study reveals that the addition of the dominant gut bacterial species *E. lenta* to a synthetic community led to significantly altered metabolomes in the mouse colon. Effects of the specific metabolites on the host remain to be determined. While the omics data provided is descriptive, the gnotobiotic setting of this study provides new insights into the causal role of *E. lenta* within the gut *in vivo*. Studying the effects of a broader range of *Coriobacteriia* species will require refined colonisation strategies. Regarding *E. lenta*, the advent of genetic tools and phages available for targeted manipulations will accelerate the pace of discoveries on its role in gut microbiome-host interactions^[78-80]^.

## DECLARATIONS

### Availability of data and materials

Raw data from the *16S rRNA* gene amplicon analysis was submitted to ENA (project PRJEB67952). The mass spectrometry proteomics data have been deposited to the ProteomeXchange Consortium via the PRIDE partner repository^[81]^ and can be accessed using the identifier PXD047016 and 10.6019/PXD047016. Metabolomics data are available at the NIH Common Fund’s National Metabolomics Data Repository (NMDR) website, the Metabolomics Workbench^[82]^ (https://www.metabolomicsworkbench.org), where it has been assigned Study ID ST003011. The data can be accessed directly via its Project DOI (http://dx.doi.org/10.21228/M8GM8G).

### Copyright

© The Author(s) 2024.
