# Supplementary material for: The human intestinal bacterium Eggerthella lenta influences gut metabolomes in gnotobiotic mice
Source: Microbiome Res Rep. 2024 Jan 18;3(2):14. doi: 10.20517/mrr.2023.65 (PMC11149096; doi:10.20517/mrr.2023.65)
Supplement: Supplementary file 1 [file mrr-3-2-14-SupplementaryMaterials.zip › mrr-3-2-14-SupplementaryMaterials/Supplementary Figures.pdf]

Supplementary Figures

The human intestinal bacterium *Eggerthella lenta* influences gut metabolomes in gnotobiotic mice

Alina Viehof[1], Sven-Bastiaan Haange[2], Theresa Streidl[1], Kristin Schubert[2], Beatrice Engelmann[2], Dirk Haller[3,4], Ulrike Rolle-Kampczyk[2], Martin von Bergen[2,5,6], Thomas Clavel[1]

[1]Functional Microbiome Research Group, Institute of Medical Microbiology, University Hospital of RWTH Aachen, Aachen 52074, Germany.
[2]Department of Molecular Systems Biology, Helmholtz Centre for Environmental Research (UFZ), Leipzig 04318, Germany.
[3]ZIEL Institute for Food and Health, Technical University of Munich, Freising 85354, Germany.
[4]Chair of Nutrition and Immunology, Technical University of Munich, Freising 85354, Germany.
[5]German Centre for Integrative Biodiversity Research (iDiv) Halle-Jena-Leipzig, Leipzig 04103, Germany.
[6]Institute of Biochemistry, University of Leipzig, Leipzig 04109, Germany.

Correspondence to: Dr. Thomas Clavel, Functional Microbiome Research Group, Institute of Medical Microbiology, University Hospital of RWTH Aachen, Pauwelsstrasse 30, Aachen 52074, Germany. E-mail: tclavel@ukaachen.de

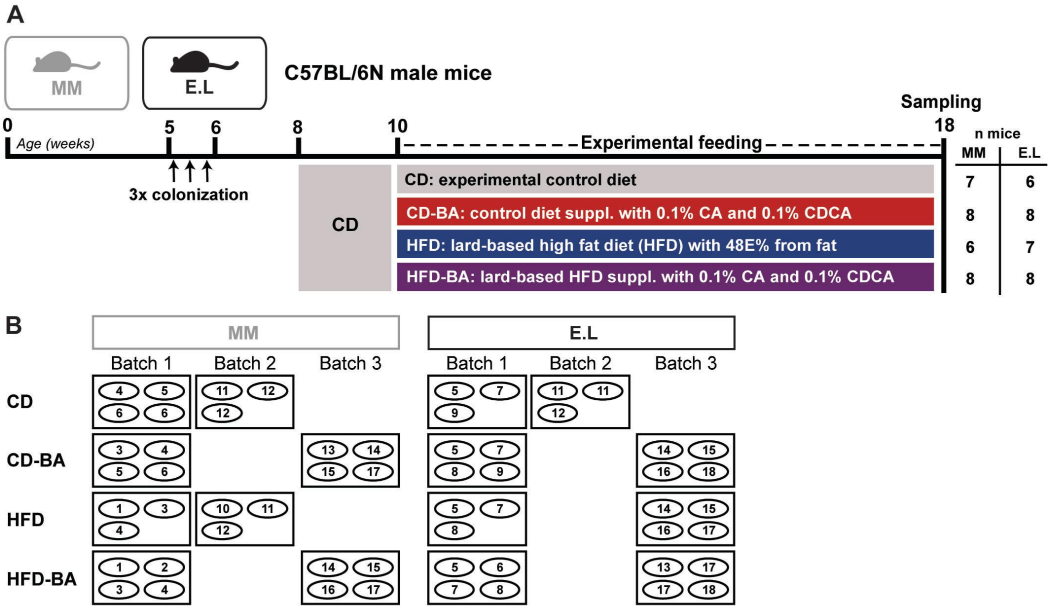

C

in percent	CD	CD-BA	HFD	HFD-BA
Ssniff ref. no.	S5745-E902	S5745-E905	S5745-E930	S5745-E935
Casein	24	24	24	24
Cornstarch	47.8	47.6	27.8	27.6
Maltodextrin	5.6	5.6	5.6	5.6
Sucrose	5	5	5	5
Cellulose	5	5	5	5
L-Cysteine	0.2	0.2	0.2	0.2
Vitamins	1.2	1.2	1.2	1.2
Minerals & trace elements	6	6	6	6
Choline chloride	0.2	0.2	0.2	0.2
Soya oil	5	5	5	5
Lard	-	-	20	20
Cholic acid	-	0.1	-	0.1
Chenodeoxycholic acid	-	0.1	-	0.1

Supplementary Figure 1. Design of the gnotobiotic experiment. (A) Germfree mice were colonised with cryo-preserved mixtures of OMM12 caecal suspension with or without *Coriobacteriia* (E.L and MM, respectively) at the age of five weeks. After two weeks of metabolic acclimatisation to the synthetic control diet (CD) (week eight to ten), the mice were fed either CD, CD supplemented with the primary bile acids cholic acid (CA) and chenodeoxycholic acid (CDCA) (each 0.1%) (CD-BA), lard-based high-fat diet (HFD), or HFD supplemented with CA and CDCA (HFD-BA) for eight weeks; (B) Distribution of the mice within the experimental groups (colonisation-diet pairs) according to cages and litters. Litters were generated in multiple batches as follows (date of first gavage): 1, 24.07.2017; 2, 14.08.2017; 3, 21.08.2017. The rectangles represent individual cages. The ovals represent individual mice; identical numbers within the ovals indicate mice that originated from the same litter (i.e., litters were divided across the experimental groups); (C) Reference and composition of the diets.

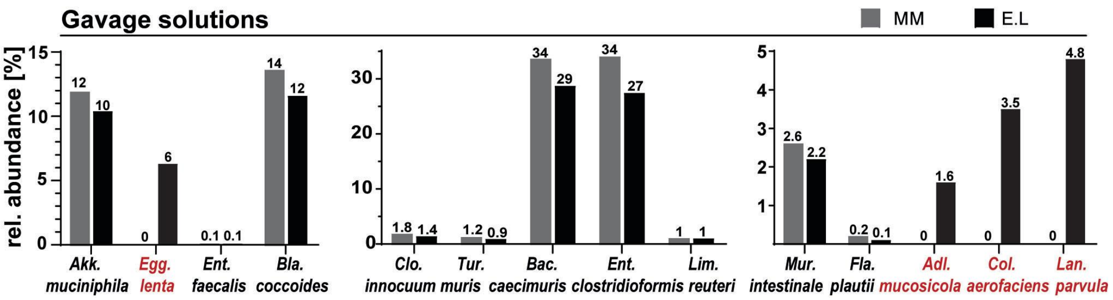

Supplementary Figure 2. 16S rRNA gene amplicon profiles of the gavage solutions. A single aliquot of each bacterial mixture (MM or E.L) used to inoculate the germfree mice was measured. Exact values for the relative abundances are written above the bars. The four *Coriobacteriia* are written in red letters.

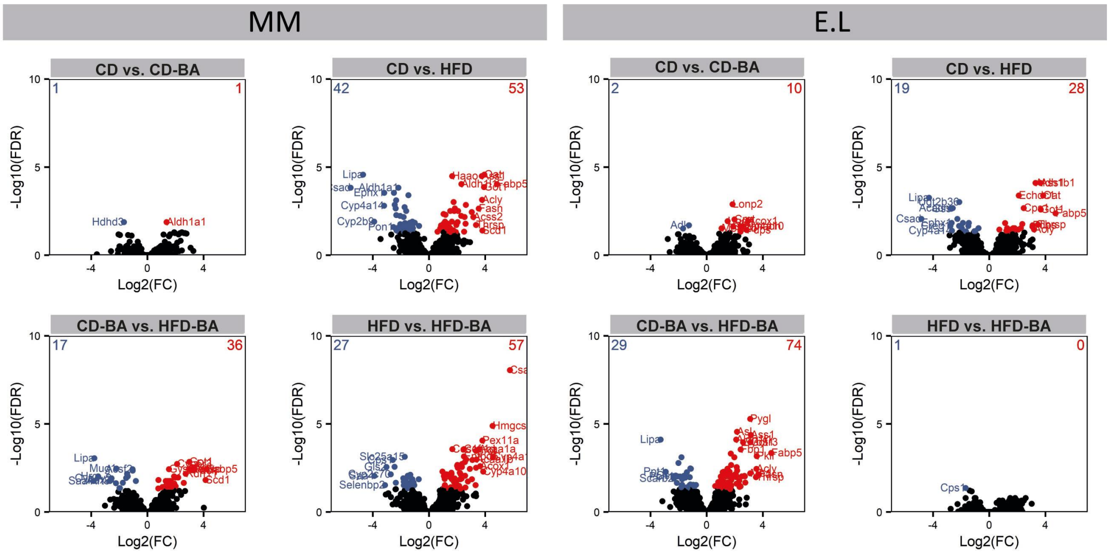

Supplementary Figure 3. Effect of the diets on liver proteomes. Volcano plots of proteins altered in mice fed with the different diets; shown for MM (left) and E.L (right) mice separately. Data on individual proteins is available in Supplementary Table 1. Abbreviations: MM, mice colonised with the mouse synthetic community OMM12; E.L, mice colonised with OMM12 and *Coriobacteriia*. CD, control diet; CD-BA, control diet supplemented with 0.2% primary bile acids; HFD, lard-based high-fat diet; HFD-BA, HFD supplemented with 0.2% primary bile acids.

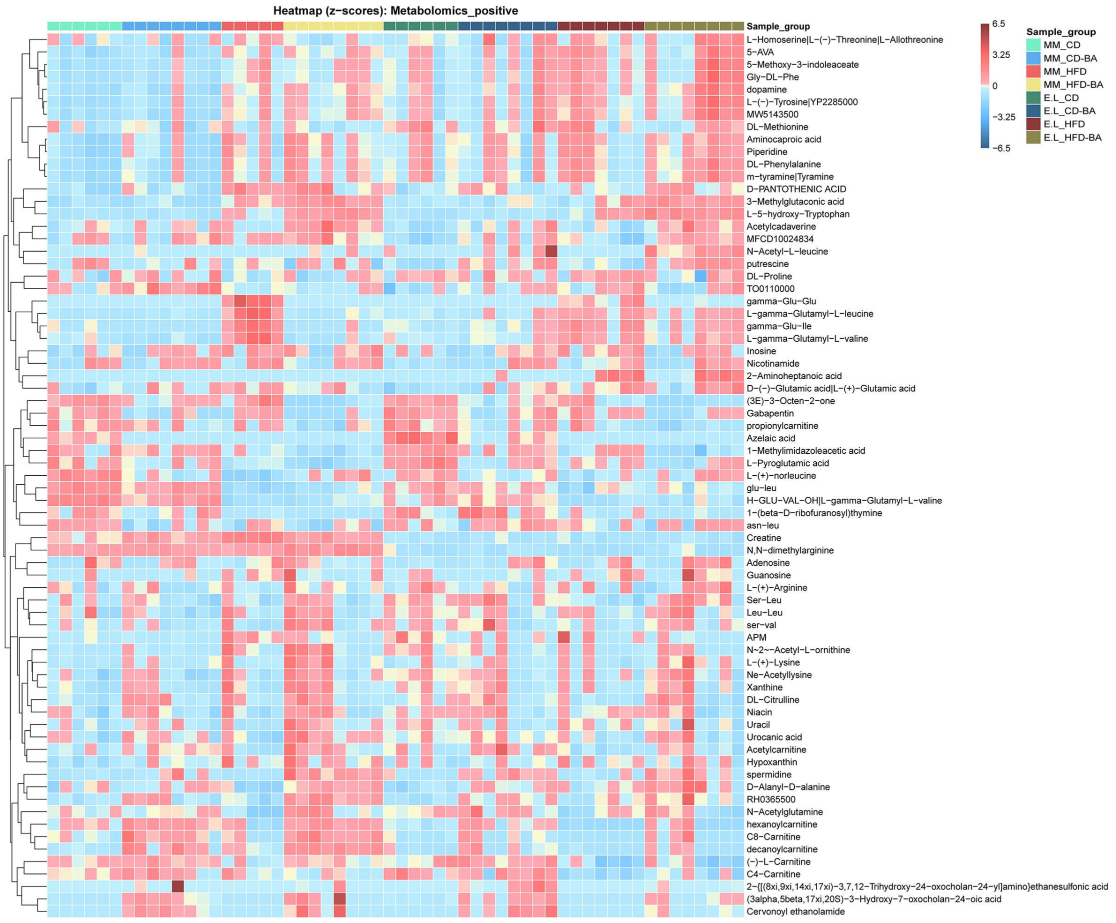

Supplementary Figure 4. Heatmap of colon metabolomes for positive ionisation mode. The colour gradient in the heatmap (from blue to red) depicts the z-scores. Each column represents one mouse and data are sorted according to colonisation/diet groups.

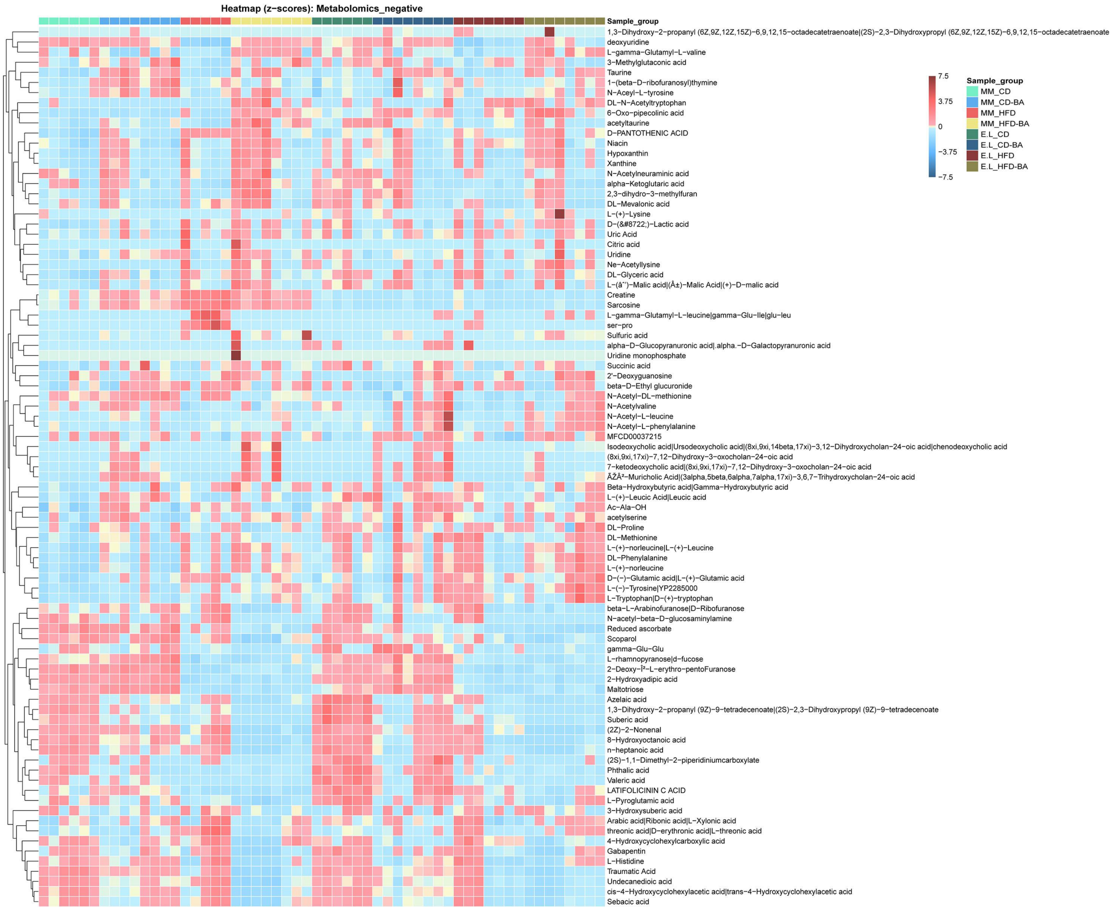

Supplementary Figure 5. Heatmap of colon metabolomes for negative ionisation mode. The colour gradient in the heatmap (from blue to red) depicts the z-scores. Each column represents one mouse and data are sorted according to colonisation/diet groups.
